# Identification and characterisation of a cryptic Golgi complex in *Naegleria gruberi*

**DOI:** 10.1242/jcs.213306

**Published:** 2018-04-09

**Authors:** Emily K. Herman, Lyto Yiangou, Diego M. Cantoni, Christopher N. Miller, Francine Marciano-Cabral, Erin Anthonyrajah, Joel B. Dacks, Anastasios D. Tsaousis

**Affiliations:** 1 Department of Cell Biology, University of Alberta, Edmonton, Alberta, Canada, T6G 2H7; 2 Laboratory of Molecular and Evolutionary Parasitology, RAPID group, School of Biosciences, University of Kent, Canterbury, Kent CT2 7NJ, UK; 3 Department of Microbiology and Immunology, Virginia Commonwealth University, School of Medicine, 1101 E. Marshall St, Richmond, VA 23298-0678, USA

**Keywords:** COPI, Protist, Evolutionary cell biology, Membrane trafficking, Brefeldin A, Dictyosome

## Abstract

Although the Golgi complex has a conserved morphology of flattened stacked cisternae in most eukaryotes, it has lost the stacked organisation in several lineages, raising the question of what range of morphologies is possible for the Golgi. In order to understand this diversity, it is necessary to characterise the Golgi in many different lineages. Here, we identify the Golgi complex in *Naegleria*, one of the first descriptions of an unstacked Golgi organelle in a non-parasitic eukaryote, other than fungi. We provide a comprehensive list of Golgi-associated membrane trafficking genes encoded in two species of *Naegleria* and show that nearly all are expressed in mouse-passaged *N. fowleri* cells. We then study distribution of the Golgi marker (*Ng*)CopB by fluorescence in *Naegleria gruberi*, identifying membranous structures that are disrupted by Brefeldin A treatment, consistent with Golgi localisation. Confocal and immunoelectron microscopy reveals that *Ng*COPB localises to tubular membranous structures. Our data identify the Golgi organelle for the first time in this major eukaryotic lineage, and provide the rare example of a tubular morphology, representing an important sampling point for the comparative understanding of Golgi organellar diversity.

This article has an associated First Person interview with the first author of the paper.

## Abstract (precis)

**Highlighted Article:** The first micrographic evidence of a cryptic Golgi organelle in the amoeba *Naegleria gruberi* is presented, revealing the Golgi in this species has a rare tubular morphology.

## INTRODUCTION

Although initially described by Camillo Golgi in 1898 as a tubular network, based on light microscopy visualisation, electron microscopy in mammalian cells and many diverse eukaryotes has revealed the Golgi complex to be a stack of flattened membranes, or cisternae. Inside these cisternae, proteins are modified via glycosylation and transported to the plasma membrane or to endolysosomal organelles. In mammalian cells, disrupting the stacked structure of the Golgi leads to numerous defects in these processes, and Golgi fragmentation is observed in autoimmune diseases, cancer, Huntington's, Parkinson's and Alzheimer's diseases (see Zhang and Wang, 2016 and references therein). However, a few examples of eukaryotes with unstacked Golgi are found across the evolutionary tree, and include the budding yeast *Saccharomyces cerevisiae* (Preuss et al., 1992), as well as parasitic taxa such as *Plasmodium falciparum*, *Entamoeba histolytica* and *Giardia intestinalis* (see Mowbrey and Dacks, 2009 and references therein).

Many of these organisms were initially thought not to possess Golgi and even to have speciated away from the rest of eukaryotes prior to the origin of the organelle. We now know that this is not the case, based on various lines of evidence. The question therefore becomes, given the conservation of stacked Golgi morphology in the vast majority of eukaryotes, what other structural diversity exists in Golgi organelles? In *S. cerevisiae*, the Golgi compartments are dispersed in the cytoplasm, appearing as puncta as determined through staining with immunofluorescent Golgi markers (Wooding and Pelham, 1998). *E. histolytica* was originally thought to not have a Golgi, but an ultrastructural study showed that this was an artefact of the fixation process for transmission electron microscopy (TEM), and presented micrographic evidence of dispersed cisternae in the cell (Chávez-Munguia et al., 2000). Finally, Golgi functions in *G. intestinalis* are stage specific, and are carried out in encystation-specific vesicles that form dispersed compartments (Stefanic et al., 2009). Unlike typical Golgi, they are not steady state organelles, but arise in response to cyst wall material formation in the endoplasmic reticulum (ER) (Marti et al., 2003a,b).

Regardless of morphology, organisms with unstacked Golgi encode the membrane trafficking factors necessary for Golgi function (Mowbrey and Dacks, 2009). An extensive set of membrane-trafficking machinery has been characterised as being involved in vesicle trafficking events to, from, and within the Golgi, with distinct paralog or complexes acting at these steps. These have been shown to be conserved across eukaryotes (Klute et al., 2011) and have been used as genomic signatures for the presence of Golgi organelles in many of the lineages thought once to lack the organelle. However, further characterisation of cryptic Golgi in evolutionarily dispersed lineages is necessary to have a better understanding of Golgi organellar evolution and the diversity of form that is possible for this organelle.

*Naegleria gruberi* is a free-living microbial eukaryote that is evolutionarily distant from animals, yeast and plants. It is commonly found in both aerobic and microaerobic soil and freshwater environments worldwide (De Jonckheere, 2002; Fulton, 1974, 1993). The closely related *Naegleria fowleri* is an opportunistic neuropathogen of humans and animals, killing ∼95% of those it infects within 2 weeks (Carter, 1970). In 2010, the *N. gruberi* genome was published, revealing a remarkably complex repertoire of cytoskeletal, sexual, signaling and metabolic components (Fritz-Laylin et al., 2010), as well as a highly complete membrane trafficking system (MTS). *Naegleria* is a member of the supergroup Excavata, which also contains the trypanosomatids, *Trichomonas vaginalis* and *Giardia intestinalis*, of parasitological importance, and the rodent gut commensal *Monocercomonoides* sp., which is both anaerobic and amitochondriate. Thus, *N. gruberi* remains one of the few free-living excavates with a complete and publicly available genome, making it a key sampling point for studying eukaryotic evolution, and potentially a useful model system for studying eukaryotic cell biology outside of the animals, yeast and plants. One distinctive cellular feature of *Naegleria* is that it lacks a visibly identifiable Golgi organelle. This is in fact a diagnostic feature of the larger taxonomic group to which *Naegleria* belongs, the heteroloboseans (Adl et al., 2012) and has been since its inception (Page and Blanton, 1985).

Despite some proposals for membranous structures as putative homologues of the Golgi (Stevens et al., 1978), the only evidence supporting the presence of the organelle in *Naegleria* has been the bioinformatically predicted Golgi-associated proteins, identified in the genome project (Fritz-Laylin et al., 2010). We here address the identification and visualisation of the Golgi structure in *Naegleria* using a multidisciplinary approach and present the first molecular and cellular evidence for the presence of a punctate Golgi in *N. gruberi*, distinct from other endomembrane organelles.

## RESULTS

### *Naegleria* encode and express Golgi-associated membrane trafficking machinery

*Naegleria*, along with the rest of the heteroloboseans, have been diagnostically described as lacking a visible stacked Golgi (Adl et al., 2012; Page and Blanton, 1985). However, sequencing and annotation of the *N. gruberi* genome suggested that it encodes many of the necessary components for Golgi function (Fritz-Laylin et al., 2010).

**Fig. 1.**
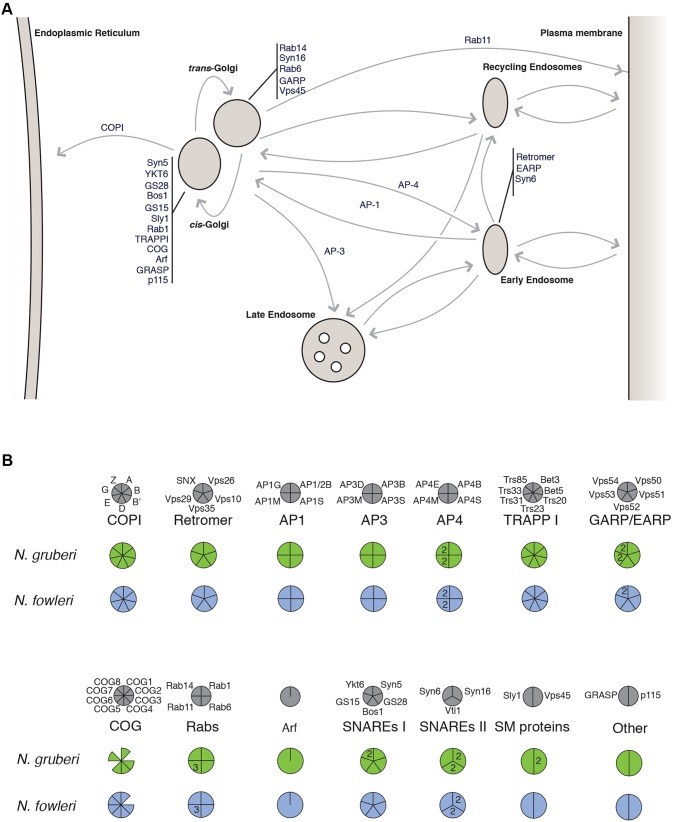
***In silico* prediction of Golgi-associated proteins in *Naegleria*.** (A) Cartoon illustrating the membrane trafficking pathways in which Golgi-associated proteins identified in *Naegleria* are known to function in canonical systems. (B) Coulson plot showing the presence of Golgi-associated proteins shown in A in *N. gruberi* and *N. fowleri*. Grey circles represent the complement of Golgi-associated proteins that are generally conserved in eukaryotes. Below, filled segments represent identified homologues, while missing segments indicate that no homologue could be found. Paralogue numbers are shown within the segments.

We first sought to expand this list in *N. gruberi*. In cases where homologous sequences could not be identified by Fritz-Laylin and colleagues (Fritz-Laylin et al., 2010), we performed additional BLAST searches using functionally characterised human sequences as queries to search the genome and predicted proteome of *N. gruberi* (Table S1). We identified several additional homologues not originally reported in the genome paper, including three members of the COG tethering complex, a nearly complete EARP/GARP complex, and a single Syntaxin 6 orthologue. As the Qa-, Qb- and Qc-SNAREs are highly paralogous gene families, phylogenetic analyses were performed in order to classify orthologues (Fig. S1A–C). Therefore, the set of Golgi-associated MTS machinery in *N. gruberi* is even more complete than previously thought (Fig. 1; Table S1).

The presence of these genes in one *Naegleria* species suggests functional relevance. Nonetheless, the possibility remains that the genes may not be translated. Examination of a publicly available transcriptome for *N. gruberi* identified expression data for 37 of the 67 genes (Table S1). However, since these are Sanger-derived expressed sequence tag (EST) data, which is not a complete reflection of all genes transcribed, there is the possibility that even more of the genes are indeed expressed. In order to determine whether these genes are present in other *Naegleria* species, and more importantly expressed, we generated transcriptomic data from *N. fowleri*. The amoeba was grown both axenically and passaged through mice, and mRNA was then extracted. Homology searching was then performed to identify Golgi-related MTS genes in the resulting transcripts, and the relative expression of these genes under each condition was calculated. We identified expressed transcripts for all 66 Golgi-associated MTS genes in *N. fowleri* (Table S1). All *N. gruberi* sequences were shown to have an orthologue in *N. fowleri*, with the exception of three *N. gruberi*-specific paralogues (Vps53A, Ykt6B and Vps45B). Furthermore, *N. fowleri* encodes and expresses two additional members of the COG complex. These results suggest that *Naegleria* not only encodes, but also expresses, Golgi trafficking and structural proteins.

### Visualisation of the Golgi in *N. gruberi*

**Fig. 2.**
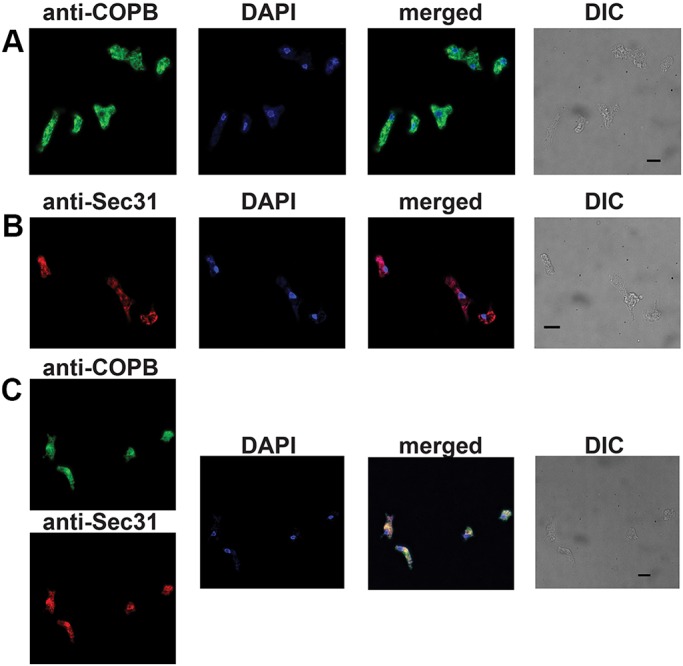
**Indirect fluorescence assay of *N. gruberi* cells.** (A) Cellular localisation of COPI in *N. gruberi* cells. Chicken anti-*N.gruberi* COPB antiserum (1:250; green) shows a discrete localisation in the cells, while DAPI stains the *Naegleria* nucleus and mitochondrial DNA. Differential interference contrast (DIC) images show the cells used for immunofluorescence. (B) Cellular localisation of Sec31 in *N. gruberi* cells. Rat anti-*N.gruberi* Sec31 antiserum (1:250; red) shows a discrete localisation in the cells, while DAPI stains the *Naegleria* nucleus and mitochondrial DNA. DIC images show the cells used for immunofluorescence. (C) Cellular localisation of COPI and Sec31 in *N. gruberi* cells. Chicken anti-*N.gruberi* COPB antiserum (green) shows a discrete localisation in the cells that is not co-localised with the rat anti-*N.gruberi* Sec31 antiserum (red), while DAPI stains the *Naegleria* nucleus and mitochondrial DNA. DIC images show the the cells used for immunofluorescence. The experiment was performed three times in six replicates. Scale bars: 10 μm.

A commonly used marker for Golgi in eukaryotic cells is the COPI complex, which forms vesicle coats for trafficking from the *cis*-Golgi to the ER (Orci et al., 1986; Szul and Sztul, 2011), and acts at *cis* and intermediate Golgi compartments (Papanikou et al., 2015). In order to visualise the *N. gruberi* Golgi through immunofluorescence microscopy, we generated antibodies specific to the β-subunit of the COPI complex (*Ng*COPB) and to the Sec31 protein of the COPII complex (*Ng*Sec31), which is specific to the ER, as a comparison point for an endomembrane organelle of the early secretory system. The generated antisera, from chicken and rat, respectively, showed high specificity for *Ng*COPB and *Ng*Sec31 in western blots, as they recognised a protein with expected size of 114.5 kDa and 145.7 kDa in the cell lysates extracted from *N. gruberi* (Fig. S2). Immunofluorescence microscopy of *N. gruberi* cells showed distinct patterns for the two antibodies (Fig. 2). Consistent with standard ER morphology, the *Ng*Sec31 antisera showed a network-like localisation around the nucleus of the organism. By contrast, the *Ng*COPB antisera showed some cytosolic staining, consistent with COPB being a cytosolic and peripheral membrane protein, but strikingly, we see punctuated and tubular localisation around the cell. Some apparent overlap was observed, as expected for organelles that span the breadth of the cells. However, clear areas of non-overlap were seen, consistent with these structures being discrete organelles.

### Brefeldin A treatment disrupts COPB localisation

**Fig. 3.**
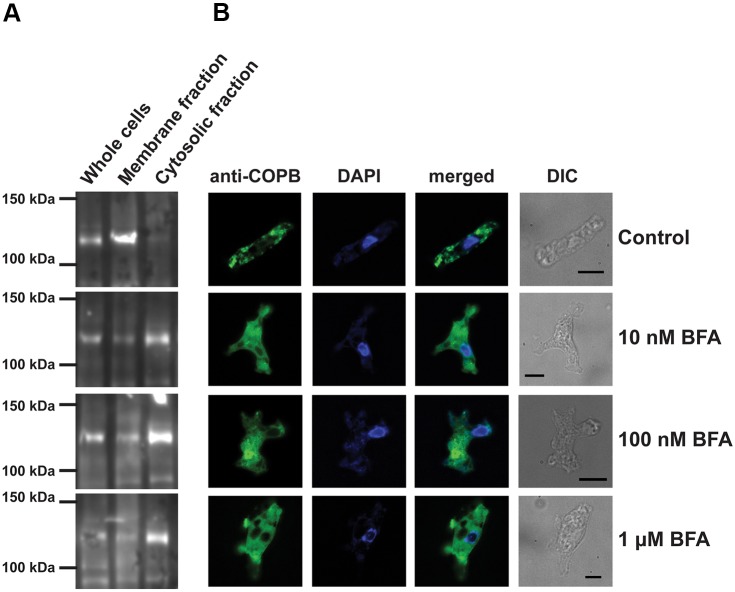
**BFA changes the localisation of COPB in *N. gruberi*.** (A) Western blot of whole-cell, membrane and cytosolic fractions using the anti-COPB antisera, in the presence of various concentrations or absence of BFA. Upon treatment with increased concentrations of BFA from 10 nM to 1 μM after 3 h of incubation, COPB intensity increases in the cytosolic fraction, while it decreases in the membrane fractions. For each experiment, we used the same initial number of *Naegleria* cells, and after a Bradford assay, the same amount of protein was loaded in the gel. (B) Indirect fluorescence assay of *Naegleria* cells after BFA treatment. Chicken anti-*N.gruberi* COPB antiserum (1:200; green) shows localisation of COPI in the cells, while DAPI stains the *Naegleria* nuclei and mitochondrial DNA. As with the western blot experiments, with an increased concentration of BFA from 10 nM to 1 μM after 3 h of incubation, COPB intensity increases in the cytosol, while the membranous localisation disappears. Differential interference contrast (DIC) images show the cells used for immunofluorescence. The experiment was performed five times in three replicates. Scale bars: 10 μm.

To further assess whether the *Ng*COPB antisera was marking Golgi organelles, we tested whether the observed discrete localisation is inhibited by Brefeldin A (BFA), a fungal metabolite that rapidly and reversibly inhibits transport of secretory proteins resulting in relocation of Golgi-resident proteins to the ER and COPB to the cytoplasm (Klausner et al., 1992). Subcellular fractionation of membrane constituents showed that treatment with BFA at 10 nM, 100 nM and 1 µM for 3 h shifted the COPB intensity from the internal membrane to the cytosolic fraction (Fig. 3A). Consistent with the previous results, immunofluorescence microscopy demonstrated that the punctuated/tubular localisation disappears as concentrations of BFA increase (Fig. 3B).

### The *Naegleria* Golgi appears as a tubular organelle

**Fig. 4.**
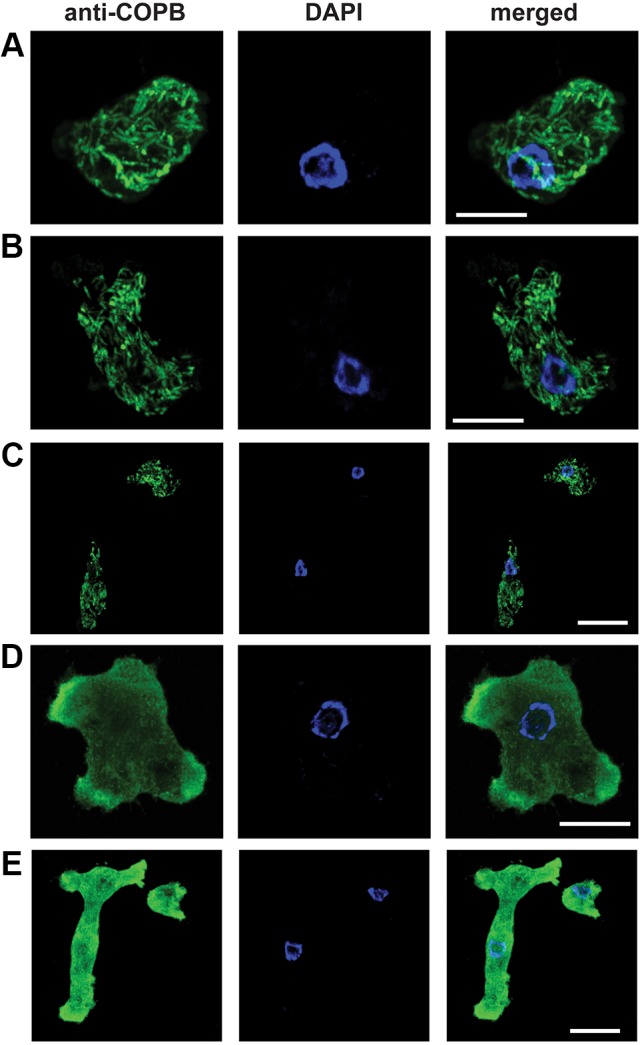
**Confocal microscopy of COPI localisation in individual *N. gruberi* cells.** (A) Cellular localisation of COPI in a *N. gruberi* cell. Chicken anti-*N.gruberi* COPB antiserum (1:250; green) shows a tubular localisation in the cell, while DAPI stains the *Naegleria* nucleus and mitochondrial DNA. The image is a result of a 3D rendering of 28 overlapping individual 0.284 μm thick sections, with a final representative thickness of 7.95 μm. Different angles of the same image can been found in Fig. S3A. (B) Cellular localisation of COPI in a *N. gruberi* cell. Chicken anti-*N.gruberi* COPB antiserum (1:250; green) shows a tubular localisation in the cell, while DAPI stains the *Naegleria* nucleus and mitochondrial DNA. The image is a result of a 3D rendering of 16 overlapping individual 0.295 μm thick sections, with a final representative thickness of 4.624 μm. Different angles of the same image can been found in Fig. S3B. (C) Cellular localisation of COPI in two *N. gruberi* cells. Chicken anti-*N.gruberi* COPB antiserum (1:250; green) shows a tubular localisation in the cell, while DAPI stains the *Naegleria* nuclei and mitochondrial DNA. The image is a result of a 3D rendering of 21 overlapping individual 0.284 μm thick sections, with a final representative thickness of 5.96 μm. Different angles of the same image can been found in Fig. S3C. (D) Cellular localisation of COPI in a *N. gruberi* cell. Chicken anti-*N.gruberi* COPB antiserum (1:250; green) shows a cytosolic localisation in the cell after treatment with 10 nM of BFA for 3 h, while DAPI stains the *Naegleria* nucleus and mitochondrial DNA. The image is a result of a 3D rendering of 32 overlapping individual 0.29 μm thick sections, with a final representative thickness of 9.27 μm. Different angles of the same image can been found in Fig. S3D. (E) Cellular localisation of COPI in two *N. gruberi* cells. Chicken anti-*N.gruberi* COPB antiserum (1:250; green) shows a tubular localisation in the cells after treatment with 1 μM of BFA for 3 h, while DAPI stains the *Naegleria* nuclei and mitochondrial DNA. The image is a result of a 3D rendering of 26 overlapping individual 0.29 μm thick sections, with a final representative thickness of 7.53 μm. Different angles of the same image can been found in Fig. S3E. The experiment was replicated five times. Scale bars: 10 μm.

We performed localisation experiments captured with confocal microscopy in order to better assess the ultrastructure of the *Naegleria* Golgi. Using the same antibody concentrations as above, we captured the localisation of COPB in various cells. 3D renderings of several sections per cell demonstrate a discrete tubular organisation (Fig. 4A–C; Fig. S3A–C, Movies 1–3), which was validated in 24 independent experiments using various antibody concentrations. This localisation pattern disappeared when the cells were incubated with various concentrations of BFA for 3 h; COPB was clearly localised in the cytosol of the *Naegleria* (Fig. 4D,E; Fig. S3D,E, Movies 4 and 5). Both localisation patterns were unlike the one seen for *Ng*Sec31 (Fig. S4).

**Fig. 5.**
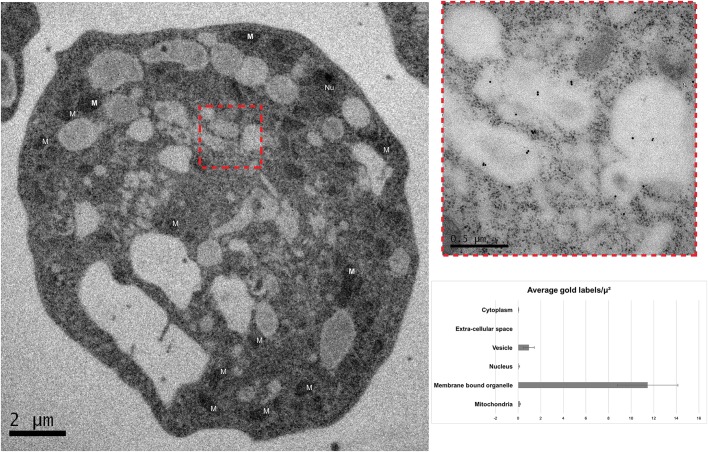
**Immuno-gold localisation of COPI in *N. gruberi* cells.** Immuno-gold localisation of COPI in an *N. gruberi* cell (TEM) shows localisation associated with membrane-bound organelles. The inset shows a higher magnification image of the organelles. Four additional images can be found in Fig. S5. The graph demonstrates the densities of labelling in the different compartments of *N. gruberi* cells, suggesting that COPI is mainly localised in the membrane bound organelles of the cell. Both mitochondria (M) and Nucleus (Nu) are indicated in the figure. The experiment was replicated three times (mean±s.d. of 60 replicates).

Finally, to investigate the subcellular localisation of *Ng*COPB even further, we utilised transmission electron microscopy (Fig. S5A,B) followed by immuno-gold electron microscopy (Fig. 5, Fig. S5C–F), which showed, with high confidence, localisation of this protein in distinct membrane organelles (1–4 µm in length) as opposed to the cytosol, nucleus, larger membrane organelles and membrane vesicles.

## DISCUSSION

In this study, we have employed a comprehensive set of tools to first identify the cryptic Golgi organelle in *Naegleria* and then investigate its ultrastructure*.* Prior to this study, the existence of a Golgi complex in *Naegleria* had only been indirectly inferred through its phylogenetic relationship with stacked Golgi-possessing lineages and, slightly more directly shown by the presence of genes in the genome of a single species whose products localise to the Golgi in canonical systems. We therefore sought to provide a more direct and convincing array of evidence. Additional homology searching within *N. gruberi* allowed the identification of several previously unreported proteins that are characteristically associated with the Golgi complex, while transcriptomic analysis of a second species demonstrated their presence in another *Naegleria* representative (*N. fowleri*) and more importantly, that these genes are indeed expressed. The complement of *Naegleria* Golgi genes suggests both *cis*-Golgi and *trans-*Golgi network (TGN) functions and retrograde pathways from the *cis*-Golgi to ER, as well as multiple routes from the TGN outward. This is consistent with complex Golgi function in *Naegleria* and fits well with other reports of vesicle coat complexity (Fritz-Laylin et al., 2010; Hirst et al., 2014).

As COPI is a well-established marker for the Golgi (Luján et al., 1995; Marti et al., 2003a), we generated a homologous polyclonal antibody against the entire *Ng*COPB protein and we used it for subsequent localisation studies. COPB is a peripheral membrane protein that translocates to membranes from the cytosol during vesicle formation for the transport of material from *cis*-Golgi to the ER (Orci et al., 1986; Papanikou et al., 2015; Szul and Sztul, 2011). In the case of *N. gruberi*, our immunofluorescence microscopy studies have demonstrated a discrete tubular localisation that occupies 17% of the total cellular volume (Table S2) and does not show the same localisation patterns as DAPI, Sec31 or ER-tracker. Both confocal and immuno-electron microscopy have provided evidence of the association of this protein with membranous structures. The identity of this membrane structures as Golgi homologues was further confirmed with BFA experiments. BFA acts on the Arf-GEF GBF1, blocking COPI vesicle formation (Klausner et al., 1992). Exercising both biochemical and microscopical means, we have demonstrated that BFA observations are consistent with the behaviour of Golgi markers in other organisms with unstacked Golgi (e.g. *Plasmodium*, *Entamoeba* and *Giardia*) upon BFA treatment (Luján et al., 1995; Manning-Cela et al., 2003; Struck et al., 2005).

Based upon the sum of our data, we conclude that the Golgi in *Naegleria* takes the form of discrete tubular compartments that do not exceed 1 µm in diameter and 4 µm in length. These tubules did not appear concentrated in any specific region of the cell, but were dispersed throughout.

There are several intriguing avenues for future investigation of *Naegleria* Golgi. Our data address only a *cis*-Golgi marker, leaving open the question of TGN organisation. We observed the Golgi in the most common life-stage of *Naegleria*, the trophozoite, but the behaviour of the organelle in the flagellate and cyst forms would be worthy of enquiry. Finally, organellar dynamics would be fruitfully investigated by using live-cell imaging and higher-resolution microscopy, such as light-sheet technologies. In *Giardia* and yeast, there is evidence of transient communication intermediates (Stefanic et al., 2009; Szul and Sztul, 2011), either tubular or vesicular, between the compartments. Understanding the mechanism of material transfer between *Naegleria* Golgi will be an exciting challenge. All of these aspects would be greatly facilitated by additional molecular cell biological tools in *Naegleria* that are currently being developed (https://www.protocols.io/view/transfection-of-naegleria-gruberi-hpub5nw). Given the complexity of the metabolic and cell biological complement encoded in its genome (Fritz-Laylin et al., 2010), we suggest that *Naegleria* is a promising model organism for comparative eukaryotic cell biology.

In theory, there is an array of forms that a stacked Golgi could take upon an evolutionary reorganisation in a lineage. These include a single large (but unstacked) organelle, a vesicular tubular network, a tubular network or discrete dispersed smaller compartments. While a single perinuclear organelle is reported in *Plasmodium* (Struck et al., 2005), to our knowledge its ultrastructure has not been examined at the electron microscopy level. The organelles of *Giardia* (Stefanic et al., 2009), *Entamoeba* (Chávez-Munguia et al., 2000), *Saccharomyces* (Wooding and Pelham, 1998) and most recently *Mastigamoeba balamuthi* (Barlow et al., 2018) are best described as discrete dispersed small compartments. By contrast, the Golgi in microsporidian lineages is reported as either an array of lamellar membranes in the cysts or as a tubular (but not vesicular) network (Beznoussenko et al., 2007; Takvorian et al., 2013). This latter organisation is most similar to that which we observe in *Naegleria*. From this diversity of form, it appears that there are no obvious constraints excluding any of the potential organellar morphologies that Golgi may take upon leaving the otherwise nearly ubiquitous stacked organisation. It will be exciting to investigate these questions in additional taxa, and by using sophisticated cell biological techniques, to further elucidate the ultrastructure of these compartments. This also raises the fundamental question of why stacking is so pervasive amongst eukaryotes, given the demonstration that other morphologies can readily exist.

## MATERIALS AND METHODS

### Comparative genomics

For all Golgi-associated MTS genes that were not identified by Fritz-Laylin and colleagues (Fritz-Laylin et al., 2010), the functionally characterised human orthologue was used as a BLASTP or TBLASTN query (Altschul et al., 1997) to search the *N. gruberi* NEG-M (Joint Genome Institute, http://genome.jgi.doe.gov/Naegr1/Naegr1.home.html) predicted proteome, EST cluster consensi and scaffolds. *N. gruberi* sequences retrieved with an E-value of 0.05 or less were used to reciprocally BLAST the *H. sapiens* and non-redundant protein databases (NCBI, https://www.ncbi.nlm.nih.gov/). To be considered true orthologues, they must retrieve the initial query or a clear orthologue with an E-value less than the 0.05 cut-off. Comparative genomics searches of the *N. fowleri* transcriptome were performed using the *N. gruberi* Golgi-associated MTS sequences as queries, or the human orthologue in cases where the *N. gruberi* sequence could not be identified, with the same criteria as above.

### Phylogenetics

Bayesian and Maximum-Likelihood phylogenetics analyses were performed to assign orthology to sequences in the Qa- and Qb-SNARE families, and the Qc-SNARE subfamily including Syntaxin 6, Syntaxin 8 and Syntaxin 10. Phylogenetically characterised sequences from *H. sapiens*, *Arabidopsis thaliana* and *Trypanosoma brucei* were aligned with *N. gruberi* and *N. fowleri* sequences identified in this study with MUSCLE v.3.8.31 (Edgar, 2004). Alignments were visualised in Mesquite v.3.03 (http://mesquiteproject.org), and manually masked and trimmed to remove positions of uncertain homology. ProtTest v3.4 (Darriba et al., 2011) was used to determine the best-fit model of sequence evolution, which was LG+G+F for the Qa- and Qb-SNARE alignments, and LG+G for the Syntaxin 6, 8 and 10 alignment. Phylobayes v4.1 (Lartillot et al., 2009) and MrBAYES v3.2.2 (Ronquist et al., 2012) programs were run for Bayesian analysis and RAxML v8.1.3 (Stamatakis, 2014) was run for maximum-likelihood analysis. Phylobayes was run until the largest discrepancy observed across all bipartitions was less than 0.1 and at least 100 sampling points were achieved, MrBAYES was used to search treespace for a minimum 10^6^ Markov chain Monte Carlo (MCMC) generations, sampling every 1000 generations, until the average standard deviation of the split frequencies of two independent runs (with two chains each) was less than 0.01. Consensus trees were generated using a burn-in value of 25%, well above the likelihood plateau in each case. RAxML was run with 100 pseudoreplicates (Edgar, 2004).

### Transcriptomics

*N. fowleri* (Ax) V212 were grown axenically at 37°C in Oxoid medium in T75 culture flasks (Cline et al., 1983). A mouse-passaged strain of V212 was obtained by intranasal inoculation of the amoebae in B_6_C_3_F_1_ mice. Mice were euthanized when symptoms of infection were evident. Care of animals was in compliance with the standards of the National Institutes of Health and the Institutional Animal Care and Use Committee at Virginia Commonwealth University. The amoebae were harvested from brain tissue and then continuously passaged through mice two times (MP2), four times (MP4) and six times (MP6). Amoebae were subsequently cultured as above for 7–10 days to remove residual brain tissue, and then RNA was extracted and converted into cDNA with the Affymetrix/USB M-MLV (cat. 78306) kit using standard protocols. Illumina libraries were constructed using the Illumina Nextera XT Workflow and sequenced in an Illumina MiSeq at the TAGC facility (UAlberta).

Between 2.8 million and 4.0 million paired-end 300 bp reads remained after pre-processing with Trimmomatic v0.36 (Bolger et al., 2014) using the arguments SLIDINGWINDOW:50:30 TRAILING:20. Reads were aligned to an unpublished genome of *N. fowleri* strain V212 produced as part of an on-going project (E. Herman et al., unpublished) using the program Tophat v2.0.10 (Trapnell et al., 2009). Transcripts were assembled for each condition using Cufflinks v2.1.1 and then merged with Cuffmerge (Trapnell et al., 2010). Cuffdiff v2.1.1 (Trapnell et al., 2013) was then used to map the reads to the merged transcripts and determine the relative expression. For some Golgi-associated MTS genes, the merged transcript appeared to be partial or incorrectly fused with another gene product, based on comparison with the *N. gruberi* homologues. To more accurately assess the expression of these transcripts, the transcript boundaries were manually modified to correct for this before mapping the reads. All newly generated *N. fowleri* gene sequences have been deposited in Genbank (https://www.ncbi.nlm.nih.gov/genbank/) as accessions MG880226–MG880243.

### *Naegleria* cell culturing

*Naegleria gruberi* strain NEG-M (kindly provided by Lillian Fritz-Laylin, Biology Department at the University of Massachusetts, Amherst, USA) was grown axenically at 28°C in M7 medium (Fulton, 1974). Cells were passaged every 3–5 days depending on their density (Fulton, 1974). Cells were passaged every 3–5 days depending on their density.

### RNA extraction

Total RNA extraction from trophozoites of *Naegleria gruberi* was performed by using an RNeasy Midi Kit (Qiagen) according to the manufacturer's protocol. cDNA was amplified according to the manufacturer's guidelines using the SuperScript III RT Reaction (Invitrogen).

### Generation of antisera to *N. gruberi* proteins

The entire *N. gruberi* NEG-M strain open reading frames (ORFs) of COPB (XP_002673194) and Sec31 (XM_002669379) were PCR amplified from the cDNA using the primers shown in Table S3 and subsequently cloned into pET16 or pET30b (Novagen), and sequenced and transformed into *Escherichia coli* BL21(DE3) PLyS cells. The expressed proteins were purified using a Ni-NTA column under native conditions. The proteins were further purified by gel electrophoresis, and 4 mg of each purified protein was used to make chicken and rat polyclonal antisera, respectively (two animals per protein; Davids Biotechnologie GmbH, Germany).

### Cell fractionation of *Naegleria*

*N. gruberi* cellular fractionation was achieved using differential centrifugation of the cell homogenate (passed five times through a gauge 33 hypodermic needle). All steps were carried out at 4°C and in the presence of the protease inhibitors (Complete Mini EDTA-free cocktail tablets, Roche). To separate cellular fractions, the cells were centrifuged at 1000 ***g*** for 10 min, and washed and resuspended in the buffer (250 mM sucrose and 20 mM MOPS, pH 7.4). The homogenate was centrifuged at 1000 ***g*** for 10 min to remove unbroken cells. The supernatant was then centrifuged at 3000 ***g*** for 15 min to collect the pellet that contained the nuclei. The supernatant was carefully collected and centrifuged at 7000 ***g*** for 30 min to discard the mitochondrial fraction (Tsaousis et al., 2014). The membrane fraction was centrifuged at 20,000 ***g*** for 3 h and the supernatant was used as the cytosolic fraction. The separated fractions were quantified by using a Bradford assay (Bio-Rad) and then were subjected to western blot analysis.

### Western blotting

Western blots of total protein extracts from *N. gruberi* trophozoite cells were incubated with the chicken anti-Cop1 (Cop1 HEN 1–347093; 1:200) and rat anti-Sec-31 (Sec31 RAT 1–347091; 1:200) antisera, followed by secondary anti-chicken and anti-rat antibodies respectively conjugated to peroxidase (Sigma). The blots were developed using the ECL protocol (Amersham) and visualised using the Syngene G:BOX XT4 machine on the GeneSys software.

### Immunofluorescence microscopy

*N. gruberi* cells were seeded at 30,000 cells/ml on NUNC LabTek Chamber slides prior to the experiment and grown for 24 h. Cells were incubated for 20 min with the ER-tracker blue-white DPX marker (Molecular Probes) and then fixed with 2% formaldehyde followed by permeabilisation with 0.1% Triton-X in 1× PBS. After blocking for 1 h in 3% bovine serum albumin (BSA) 1× PBS, the cells were probed with the chicken anti-COPI (HEN 1–347093; 1:250) and rat anti-Sec (RAT 1–347091; 1:250) antisera. Secondary Alexa Fluor 488-conjugated goat anti-chicken IgG (H-L), Alexa Fluor 488-conjugated chicken anti-rat IgG and Alexa Fluor 594-conjugated donkey anti-rat IgG (Molecular Probes) were used at a dilution of 1:1000. Cells were mounted with DAPI-containing anti-fade mounting reagent (Vectashield), and observed under an Olympus IX81 fluorescence microscope and a laser scanning Zeiss LSM 880 confocal microscope. Images were collected using Micromanager 1.4 software for fluorescence and Zeiss Zen software for confocal microscope and processed with ImageJ.

### Fixation of cells for electron microscopy and immuno-gold labelling

Aspirated cultures of *N. gruberi* were fixed for 1 h in freshly prepared PBS solution containing either 2.5% glutaraldehyde or 4% formaldehyde, for contrast transmission electron microscopy (CTEM) or immuno-electron microscopy (IEM), respectively. Both sample preparations were then washed several times with PBS. CTEM samples were stained and post-fixed with 1% osmium. Both sample preparations were then dehydrated through an ethanol series (30%, 50%, 70%, 90% and three times in 100%). The ethanol was then aspirated and replaced with the appropriate resin mixture. CTEM samples were suspended in propylene oxide (PO, Agar Scientific) and mixed thoroughly by means of a rotor; the PO was then replaced by a 50:50 mix of PO and Agar Scientific low viscosity (LV) resin and mixed again. The PO:LV resin mix was then replaced with 100% LV resin. IEM samples were similarly suspended in LR white resin (Agar Scientific). Resin permeation was aided by placing the samples in a vacuum for 2 min. The resin was then aspirated and replaced with fresh resin and the samples transferred into Beem (for CTEM, Agar Scientific) or gelatin (for IEM, Agar Scientific) capsules and hardened for 15 h in a pre-warmed 60°C oven. The hardened blocks were then polished and subsequently sectioned by ultra-microtome at a thickness of 70 µm, then placed on 50 mesh, 3 mm copper or gold EM grids for CTEM or IEM, respectively, with approximately five sections per grid. Immuno-staining of the IEM grids was performed in humidifying chambers. Blocking of the samples was achieved via a 1 h incubation in 2% BSA in PBS with 0.05% Tween 20. Primary antibody binding was performed by 15 h incubations with the primary antibody, at three dilutions (1:10, 1:50 and 1:100) at 8°C. The IEM grids were subsequently incubated for 30 min at room temperature, with the corresponding gold-conjugated secondary antibodies. Counter-staining of both CTEM and IEM sample grids was achieved via a 15 min incubation with 4.5% uranyl acetate in PBS and a 2 min incubation in Reynold's lead citrate.

### Brefeldin A experiments

Prior to each experiment (immunofluorescence microscopy or protein extraction/cell fractionation), *N. gruberi* cells were incubated at 28°C in M7 medium for 3 h with 10 nM, 100 nM and 1 μM of Brefeldin A (S7046-SEL, Stratech Scientific Ltd).

## Supplementary Material

Supplementary information
