# Supplementary material for: Identification and characterisation of a cryptic Golgi complex in Naegleria gruberi
Source: J Cell Sci. 2018 Apr 9;131(7):jcs213306. doi: 10.1242/jcs.213306 (PMC5963838; doi:10.1242/jcs.213306)
Supplement: Supplementary information [file joces-131-213306-s1.pdf]

Supplementary Figure 1A

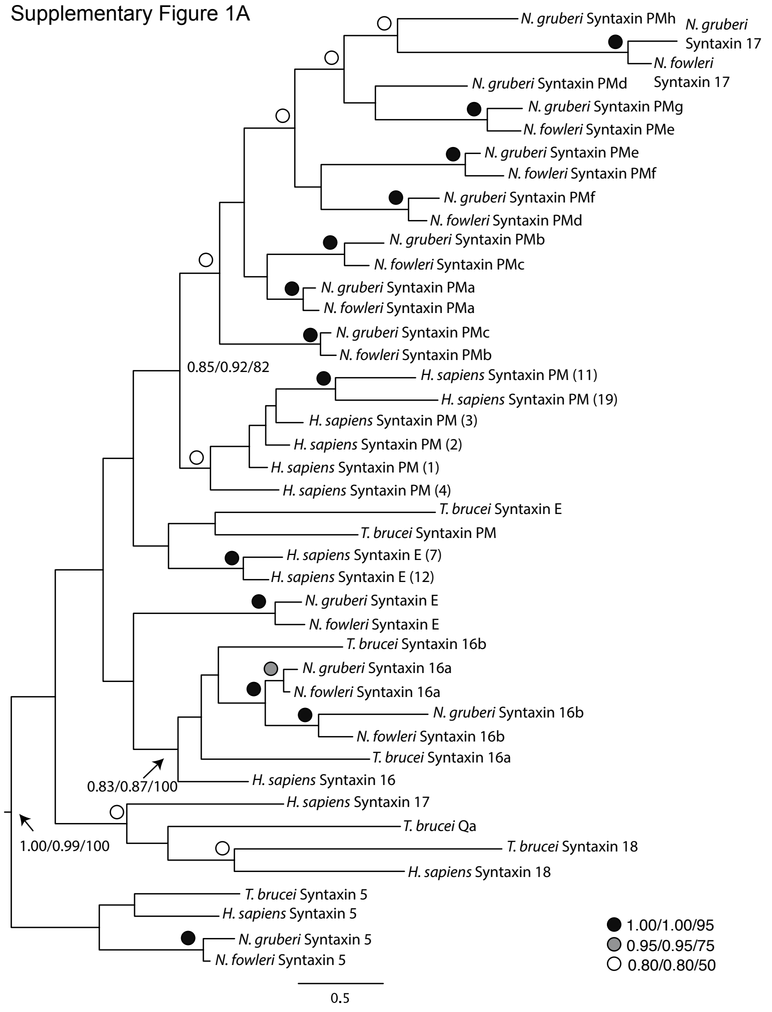

Supplementary Figure 1B

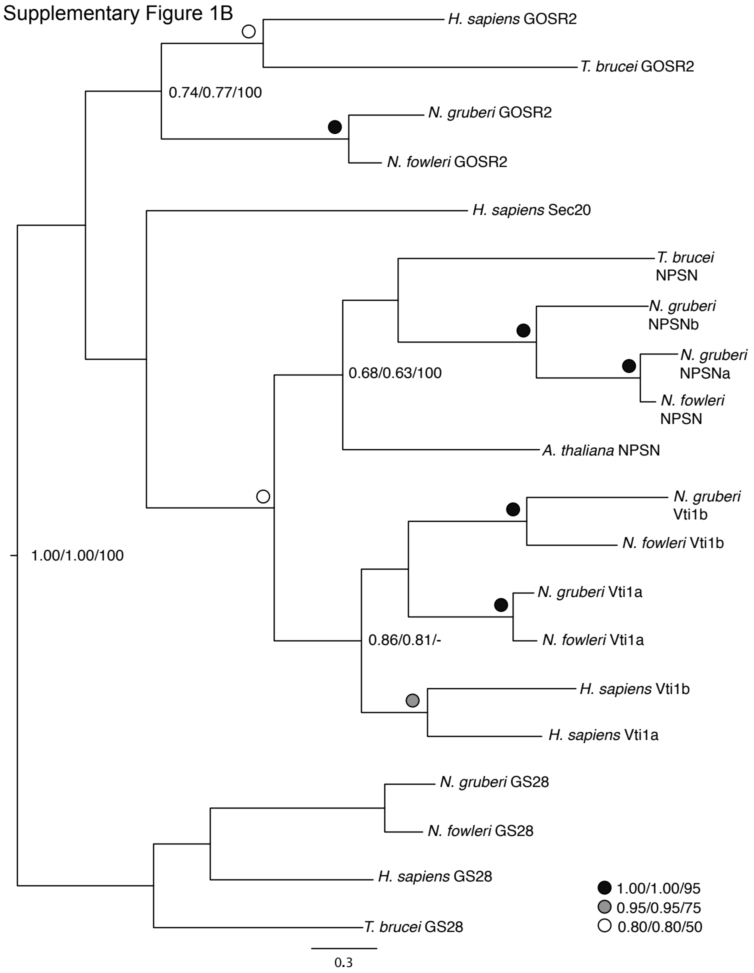

Supplementary Figure 1C

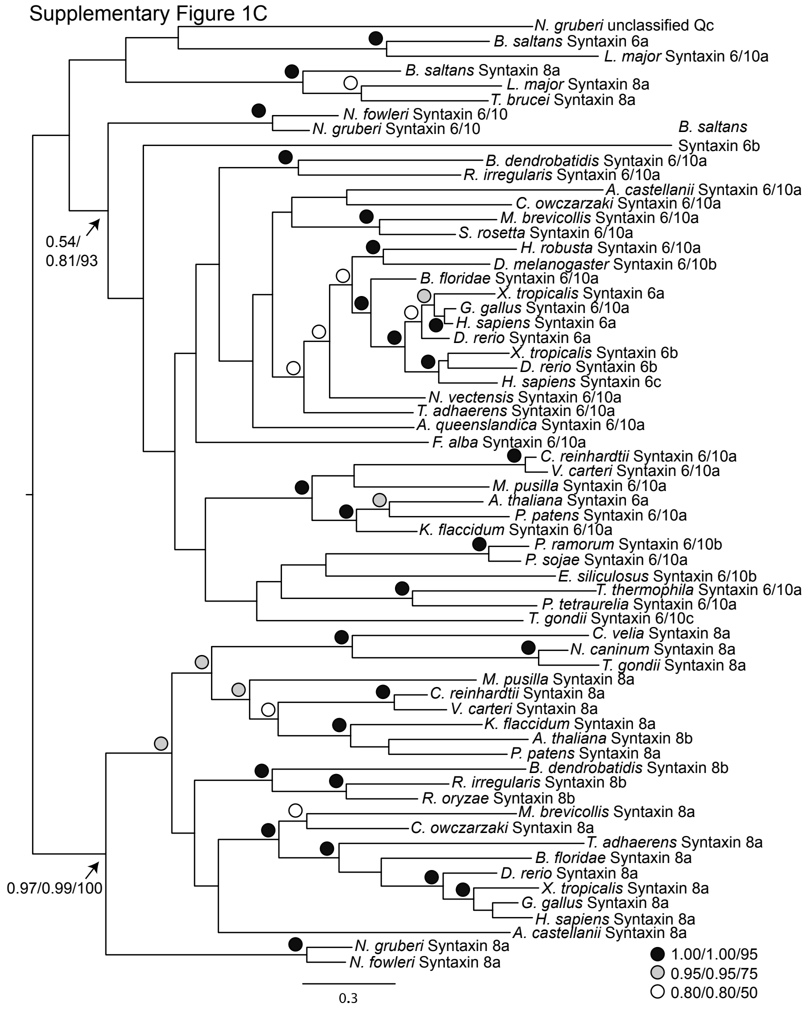

Supplementary Figure 1. Phylogenetic analysis of SNARE proteins. These phylogenies show the classification of the various Naegleria SNAREs. In each case the best Bayesian topology from MrBayes is shown with support values for subfamily-defining nodes, given as MrBayes posterior probability/PhyloBayes posterior probability/RAxML bootstrap. Other nodes are symbolized as inset. (A) Qa SNAREs, (B) Qb SNAREs, (C) Syntaxin 6/10 and Syntaxin 8 Qc SNAREs.

Journal of Cell Science • Supplementary information

Supplementary Figure 2

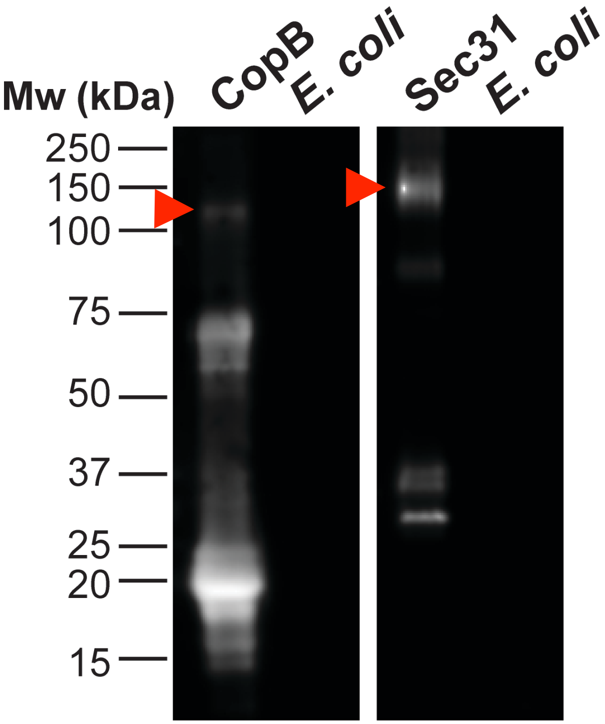

Supplementary Figure 2. Analysis of the expression of *N. gruberi* COPB and Sec31 in *E. coli* and in total protein extracts.
Western blots using the raised antisera against *Naegleria gruberi* COPB and Sec31 against the corresponding proteins extracted and purified from *E. coli*. *E. coli* proteins were used as control. First column shows the specificity of the α-COPB antisera against the heterologous expressed *N. gruberi* COPB protein at an estimated size of 117 kDa, with relevant degradation of the protein as well. The antisera does not cross-react with any of the host *E. coli* proteins, as shown in the second column. The third column shows the specificity of the α-Sec31 antisera against the heterologous expressed *N. gruberi* Sec31 protein at an estimated size of 148 kDa, with relevant degradation of the protein as well. The antisera does not cross-react with any of the host *E. coli* proteins, as shown in the fourth column.

Journal of Cell Science • Supplementary information

Supplementary Figure 3

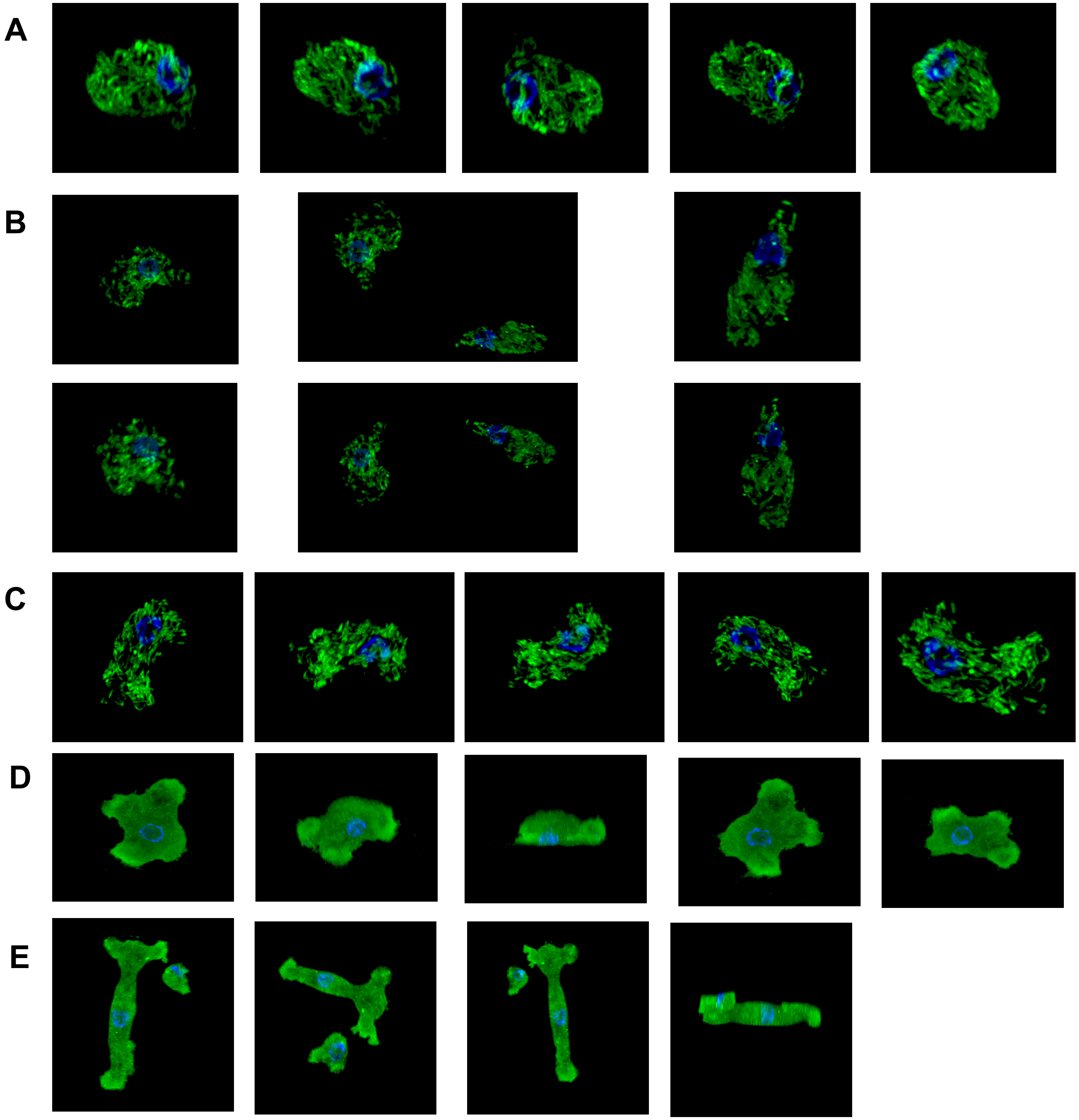

Supplementary Figure 3. Cellular staining of COPB in *Naegleria gruberi* cells using confocal microscopy
(A) *N. gruberi* 3D-stacked presentation of different perspectives displaying the localisation of COPB (green) and DAPI (blue) in a 3D rendering of 28 individual, 0.284 μm thick sections, overlapping with a final representative thickness of 7.95 μm, displayed in Fig. 4A. An animation of the cell is shown in Movie 1. (B) *N. gruberi* 3D-stacked presentation of different perspectives displaying the localisation of COPB (green) and DAPI (blue) in a 3D rendering of 16 individual, 0.295 μm thick sections, overlapping with a final representative thickness of 4.624 μm, displayed in Fig. 4B. An animation of the cell is shown in Movie 2. (C) N. gruberi 3D-stacked presentation of different perspectives displaying the localisation of COPB (green) and DAPI (blue) in a 3D rendering of 21 individual, 0.284 μm thick sections, overlapping with a final representative thickness of 5.96 μm, displayed in Fig. 4C. An animation of the cell is shown in Movie 3. D) *N. gruberi* 3D-stacked presentation of different perspectives displaying the localisation of COPB (green) and DAPI (blue) after incubation with 10 nM of Brefeldin A for three hours in a 3D rendering of 32 individual, 0.29 μm thick sections, overlapping with a final representative thickness of 9.27 μm, displayed in Fig. 4D. An animation of the cell is shown in Movie 4. (E) *N. gruberi* 3D-stacked presentation of different perspectives displaying the localisation of COPB (green) and DAPI (blue) after incubation with 1 μM of Brefeldin A for three hours in a 3D rendering of 26 individual, 0.29 μm thick sections, overlapping with a final representative thickness of 7.53 μm, displayed in Fig. 4E. An animation of the cell is shown in Movie 5.

Supplementary Figure 4

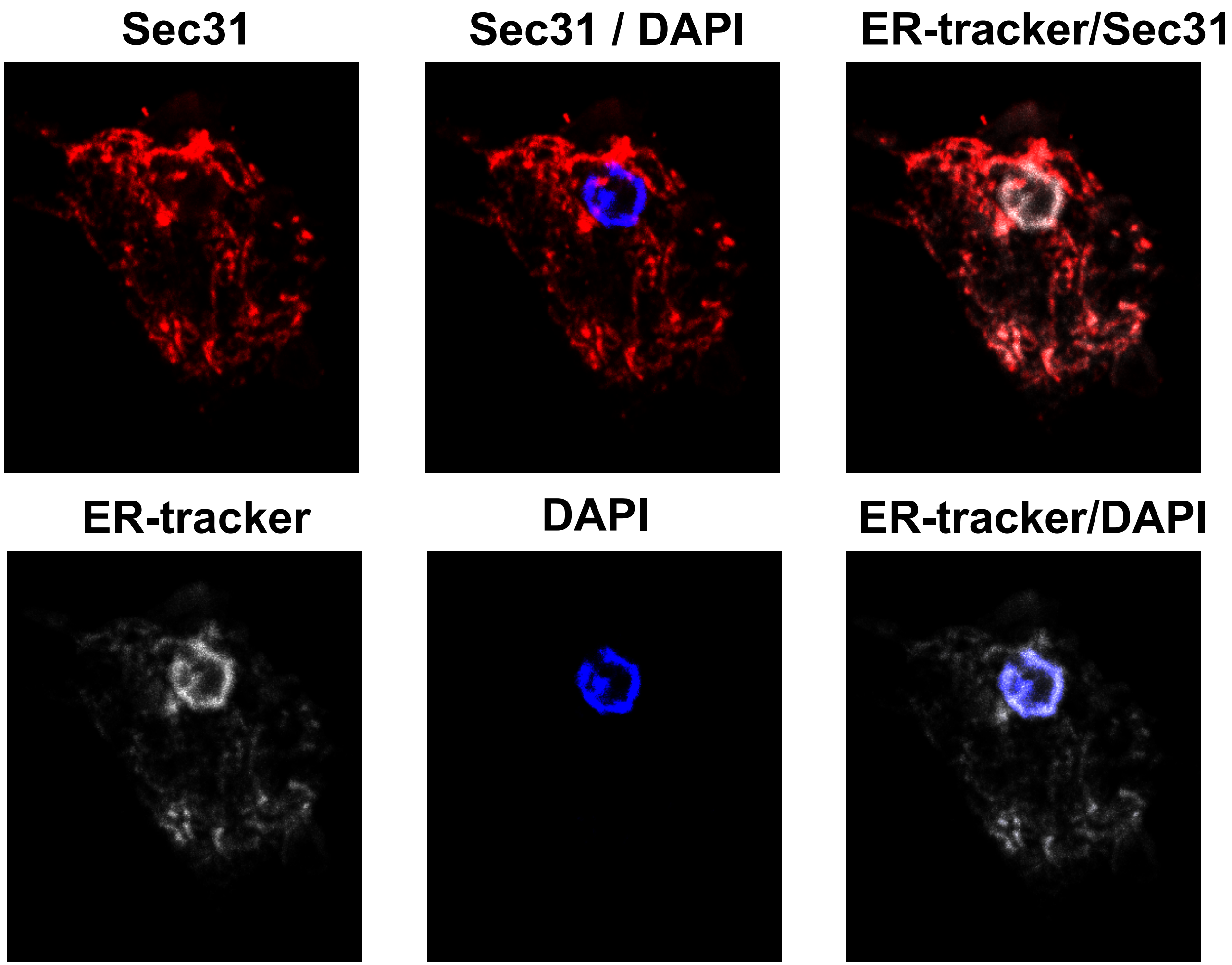

Supplementary Figure 4. Cellular staining of Sec31 in *Naegleria gruberi* cell using confocal microscopy
Confocal microscopy showing the localisation of Sec31 (red), ER-Tracker (white) and DAPI (blue) in a 3D rendering of 25 individual, 0.2 µm thick sections, overlapping with a final representative thickness of 5 µm. The images show partial co-localisation of Sec31 with ER-tracker, suggesting that the antibody recognises *N. gruberi*'s ER structure. The DAPI staining forms a ring around the nucleus, potentially due to the cells becoming stressed during the fixation procedure. The DAPI stained region is superimposed on the ER tracker marker in this region as a result to similar excitation/emission patterns between the two dyes

Supplementary Figure 5

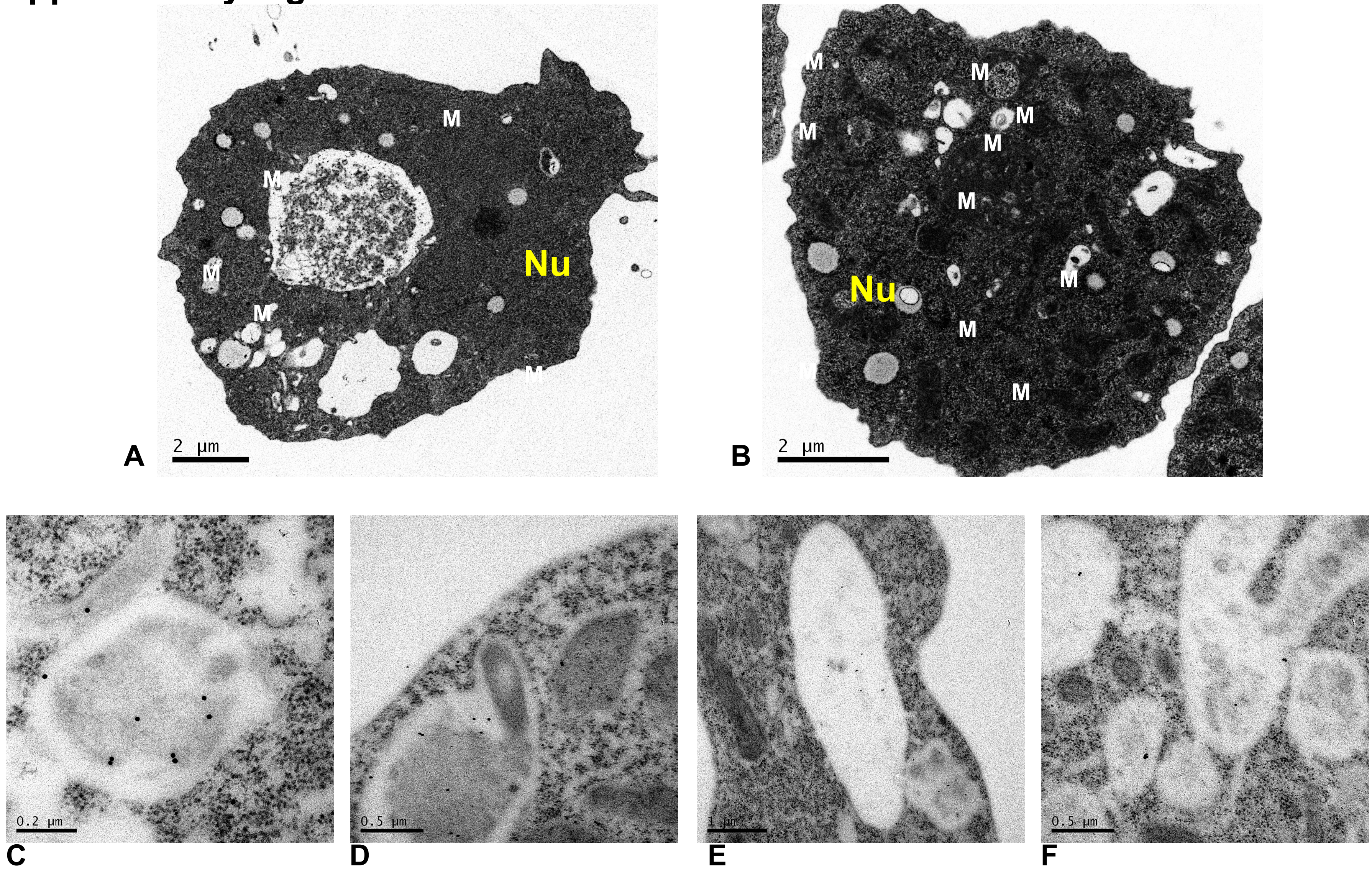

Supplementary Figure 5: Transmission-Electron Microscopy showing the cellular geography of *N. gruberi* cells
(A-B) Microscopy of *N. gruberi* cells fixed under osmium (see methodology) in order to visualize the cellular geography of the cells. Nucleus (Nu), Mitochondria (M) are labelled in the figure. Under these conditions and without immune-localisation techniques, it was difficult to identify the Golgi apparatus in these cells. (C-F) Additional images showing localisation of COPB in *Naelgeria gruberi* in various membrane bound organelles by transmission electron microscopy. Mitochondria are marked with “M”.

Journal of Cell Science • Supplementary information

Movies

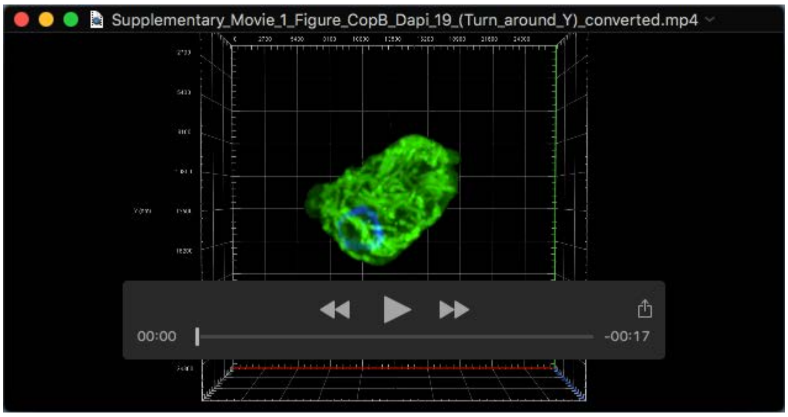

Movie 1. Animation of cellular staining of *Naegleria gruberi* cells using confocal microscopy. 3D-stacked animation displaying the localisation of COPB (green) and DAPI (blue) in a 3D rendering of 28 individual, 0.284 µm thick sections, overlapping with a final representative thickness of 7.95 µm, displayed in Fig. 4A.

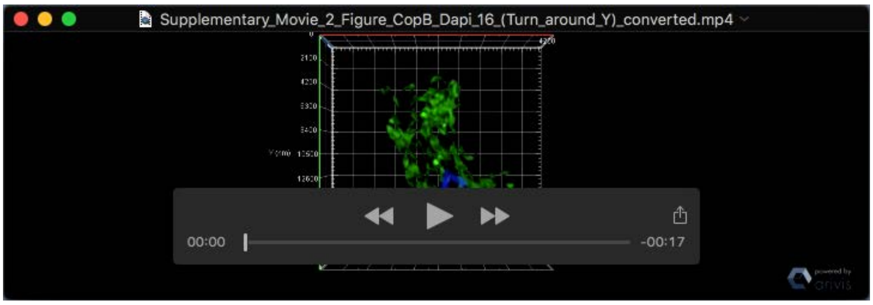

Movie 2. Animation of cellular staining of *Naegleria gruberi* cells using confocal microscopy. 3D-stacked animation displaying the localisation of COPB (green) and DAPI (blue) in a 3D rendering of 16 individual, 0.289 µm thick sections, overlapping with a final representative thickness of 4.624 µm, displayed in Fig. 4B.

Journal of Cell Science • Supplementary information

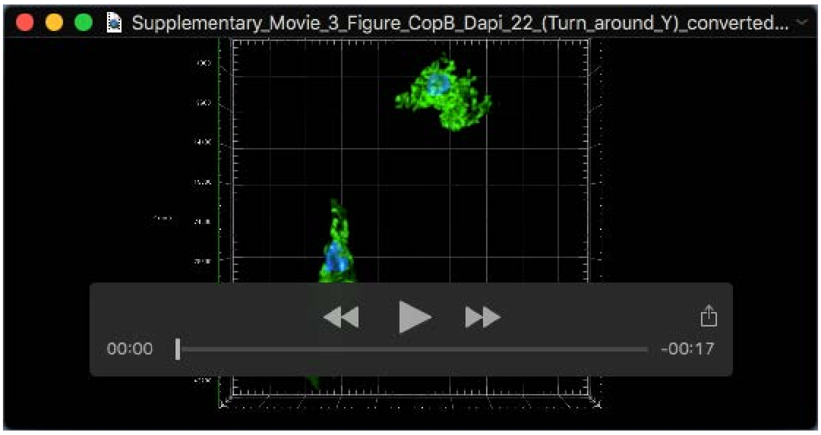

Movie 3. Animation of cellular staining of *Naegleria gruberi* cells using confocal microscopy. 3D-stacked animation displaying the localisation of COPB (green) and DAPI (blue) in a 3D rendering of 21 individual, 0.284 µm thick sections, overlapping with a final representative thickness of 5.96 µm, displayed in Fig. 4C.

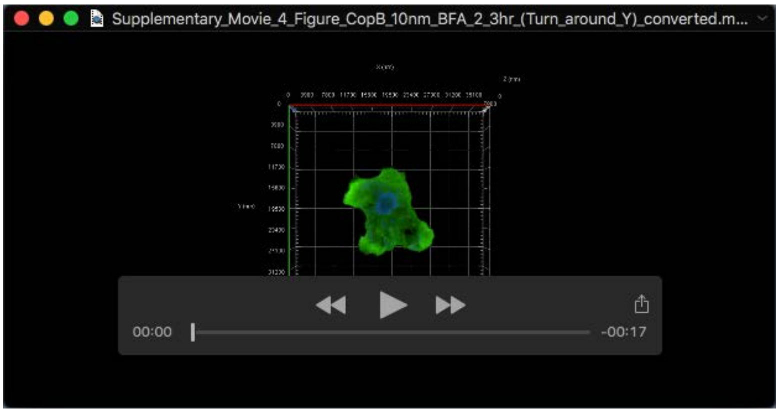

Movie 4. Animation of cellular staining of *Naegleria gruberi* cells using confocal microscopy. 3D-stacked animation displaying the localisation of COPB (green) and DAPI (blue) after incubation with 10 nM of Brefeldin A for three hours in a 3D rendering of 32 individual, 0.29 µm thick sections, overlapping with a final representative thickness of 9.27 µm, displayed in Fig. 4D.

Journal of Cell Science • Supplementary information

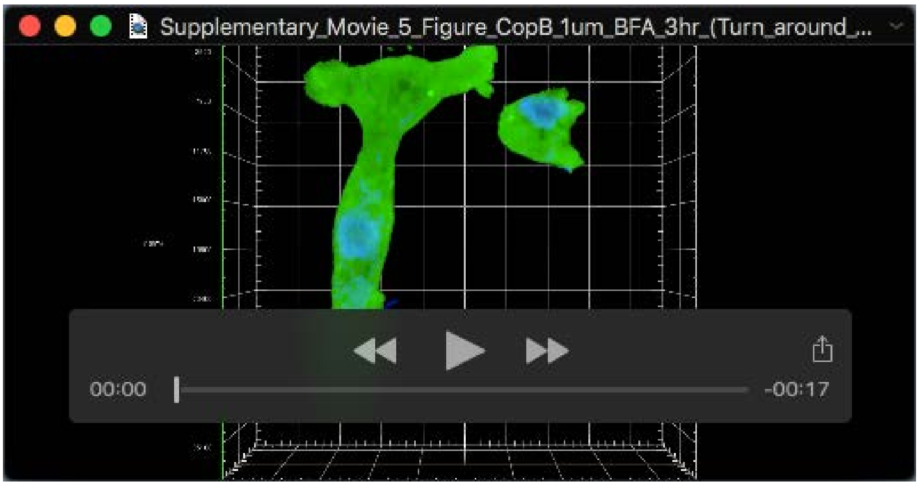

Movie 5. Animation of cellular staining of *Naegleria gruberi* cells using confocal microscopy. 3D-stacked animation displaying the localisation of COPB (green) and DAPI (blue) after incubation with 1 µM of Brefeldin A for three hours in a 3D rendering of 26 individual, 0.29 µm thick sections, overlapping with a final representative thickness of 7.53 µm, displayed in Fig. 4E.

Journal of Cell Science • Supplementary information

Table S1. Golgi-associated proteins in *N. gruberi* and *N. fowleri*, with gene expression data in axenically grown and mouse-passaged *N. fowleri*. Gene expression values given as fragments per kilobase of transcript per million mapped reads (FPKM)

Click here to Download Table S1

Table S2. Analysis data obtained from 22 individual images for areas of Golgi Bodies and Area of cells was determined using Zen Blue Software Analysis tool. First sheet presents the graph with a summary of the data and the mean calculations. The next 22 individual sheets show the data obtained from each separated image.

Click here to Download Table S2

Table S3. Primers used for cloning and sequencing genes

PRIMER NAME	SEQUENCE 5' TO 3'	RESTRICTION SITE	PURPOSE
NgCopB_BamHIF	TAT CAT ATG GCT ACA AGC TCT GCT CAT CTC	BamH1	Clone in pet16b
NgCopB_XhoIR	GAGCTCTTTCTTCAACTTCTTTGCGTCAAAC	Xho1	Clone in pet16b
NgSec31_SacIF	CCGAGCTCATGAGACTCACTTCCGTTAACAAG	Sac1	Clone in pet30b
NgSec31_NotIR	GCGCGGCCGCTTGCTTTGATGCTTTAAGACAGGTC	NotI	Clone in pet30b
NgSec31_960F	CAAGATACAGTTCTCTTGCTTGG		Sequencing
NgSec31_2401F	CACCAGATGGATTTCTGAATTCG		Sequencing
NgSec31_3025F	CAACCACTTCCAACAGTTTCCC		Sequencing
NgSec31_1783R	GCCAATCAATGGAATGGAATTTG		Sequencing
NgSyn18_BamHIF	CCGGGGATCCATGAGTGGATGGTGGGGCGTAG	BamH1	Clone in pet16b
NgSyn18_XhoIR	GAGCTCCTTAATAAGATTCTTAATGATATTAC	Xho1	Clone in pet16b

Journal of Cell Science • Supplementary information
